# Loans Versus Gifts

**Published:** 1888-01-21

**Authors:** Edmond About


### LOANS VERSUS GIFTS.

With what object has modern benevolence substituted loans to labour for gifts to idleness? It is not in order to change the form of alms, but to abolish them. The thought which inspired this generous revolution never intended retaining the poor under a skilfully-disguised form of patronage. The design is to emancipate the very persons who are helped, and to render them at once happier and more independent. In a social state, having equality as its basis, the noblest benevolence is that which permits those in want to ameliorate their own condition themselves without being indebted to anybody. —*Edmond About.*

